# Hematological Adaptations to Altitude Training in Female Water Polo Players: A Case Report of a World Championships Medal-Winning Team

**DOI:** 10.3390/sports13030086

**Published:** 2025-03-12

**Authors:** Iñigo Mujika, Jocelyn Mara, Irina Zelenkova, Rodrigo Zacca, David B. Pyne

**Affiliations:** 1 Department of Physiology, Faculty of Medicine and Nursing, University of the Basque Country, 48940 Leioa, Spain; 2 Exercise Science Laboratory, School of Kinesiology, Faculty of Medicine, Universidad Finis Terrae, Santiago 7501014, Chile; 3 Research Institute for Sport and Exercise, University of Canberra, Canberra, ACT 2617, Australia; jocelyn.mara@canberra.edu.au (J.M.); david.pyne@canberra.edu.au (D.B.P.); 4 Growth, Exercise, Nutrition and Development (GENUD) Research Group, Faculty of Health and Sport Sciences, University of Zaragoza, 50009 Zaragoza, Spain; dririnazelenkova@gmail.com; 5 Research Center in Physical Activity, Health and Leisure (CIAFEL), Faculty of Sports, University of Porto (FADEUP), 4200-450 Porto, Portugal; rzacca@fade.up.pt; 6 Laboratory for Integrative and Translational Research in Population Health (ITR), 4050-600 Porto, Portugal

**Keywords:** hemoglobin mass, blood volume, hypoxia, team sports, recovery, adaptation

## Abstract

Background: The effective monitoring of athletes’ adaptation is crucial to optimize the outcomes of altitude camps and minimize the risk of maladaptation to the hypoxic stress and intensive training. This case report assessed the hematological adaptations in 22 world-class female water polo players during a 16-day ‘live high-train high’ (LHTH) altitude camp (2320 m) and evaluated the differences between selected (n = 13) and non-selected (n = 9) players and between playing positions. Methods: Hematological parameters, including total hemoglobin mass (tHB_mass_) and blood volume, were measured before and after the camp. Resting heart rate, peripheral oxygen saturation, body mass, fatigue, and sleep were monitored daily. Results: Relative tHb_mass_ increased PRE to POST (5.4 ± 5.1%, range −3.9–20.2), but blood volume did not change (*p* = 0.797). Erythrocyte count, hemoglobin concentration, hematocrit, and red cell distribution width increased PRE–POST (*p* < 0.001, ES = 1.21–2.69), while mean corpuscular volume and hemoglobin decreased (*p* < 0.001, ES = 0.51 and 0.72, respectively). No substantial differences were observed in the hematological parameters between selected and non-selected players. There was a large difference in the change in relative blood volume between centers (n = 4, PRE 74.1 ± 5.4, POST 69.7 ± 5.9 mL/kg; mean ± SD) and field players (n = 15, PRE 80.8 ± 10.6, POST 82.8 ± 6.8 mL/kg; adj *p* = 0.046, ES = 1.15) and between centers and goalkeepers (n = 3, PRE 89.7 ± 9.6, POST 82.0 ± 7.1 mL/kg; adj *p* = 0.046, ES = 1.62). Conclusions: A 16-day LHTH camp can induce favorable hematological adaptations in world-class women’s water polo players, without substantial differences between selected and non-selected players, and larger increases in field players.

## 1. Introduction

Altitude training for elite athletes typically involves traveling to venues of increased geographical elevation, usually ranging between ~1800 and ~2500 m, to live and train for periods of 2 to 4 weeks [1,2,3,4,5]. This strategy is often used by elite endurance athletes to prepare for major international competitions. The physiological rationale for such a strategy, often termed ‘live high-train high’ (LHTH), is that the reduced barometric and partial pressure of oxygen in a hypoxic environment lowers oxygen availability, with the downstream effects of increased signaling of hypoxia-inducible factor-1α (HIF-1α), and increased erythropoietic response [6] and hemoglobin mass (tHb_mass_) [7,8]. However, non-hematological changes also induced by the hypoxic environment, such as mitochondrial gene expression and enhanced angiogenesis, glucose transport, glycolysis, and pH regulation, could enhance endurance performance [9]. Combined hematological and non-hematological adaptations, coupled with the benefits of a high-quality training camp (i.e., increased focus on training, more recovery between sessions, consistently having training partners of similar performance level, novelty of the venue, sports science support, and being away from home distractions) and the potential placebo effect of training at altitude [3] can yield an improved competition performance at sea level [1,2].

Despite the widespread popularity of LHTH among endurance athletes [4], this strategy has received much less attention among team-sport players. Detailed interest in this approach for team sports has only been highlighted by sport science research in the last decade [10,11]. An interest in natural altitude training could be less obvious for team sports, given that the physiological determinants of team-sport performance are not as well established as in individual endurance sports. Performance itself is a difficult concept to define in a team-sport context (e.g., winning vs. maintaining a high playing tempo vs. executing practiced skills under pressure) [12]. Quantifiable numerical data are precise and can indicate an athlete’s potential to perform; however, actual performance in a team-sport framework is complex and multifactorial [12]. The diverse range of team-sport training activities, the degree of inter-individual variability in the responses and adaptations to training between team members, and variable relationships between physiological qualities and performance capabilities complicate the assessment of the effectiveness of a given training strategy [11,12]. Despite these limitations, endurance and team-sport athletes share sufficient key activity profiles and energetic qualities for altitude training to be a potentially useful strategy for enhancing the physical fitness of team-sport players [10].

In view of the typical physical and physiological demands of team-sport match play, and the potential for altitude training to enhance both aerobic and anaerobic metabolism through hematological and non-hematological adaptations, it is conceivable that water polo players could reap the benefits of altitude training camps when preparing for a major tournament. Indeed, water polo is a physically and energetically demanding aquatic team sport, characterized by a highly intermittent high-intensity activity profile. During water polo match play, players typically perform ~100 high-intensity and sprint activities of ~7–14 s durations. These bursts of high-intensity activity are interspersed with periods of lower intensity activities, with a typical work-to-rest ratio within play of 1:1.6 [13,14]. In addition, water polo players are required throughout a game to swim at various intensities, tread water, wrestle with opponents, lunge in offense and defense, and pass, receive, and shoot the ball [15]. Positional differences are evident in the match activity profile, with a greater contribution of high-intensity swimming for field players, a larger emphasis on wrestling and fighting for position for center forwards, and activities alternating between easy-sculling, ready sculling, hands-up position, jumping, and passing for goalkeepers [13,16]. Although these activity profiles indicate that, irrespective of playing position, both aerobic and anaerobic metabolic pathways are heavily taxed during water polo match play [17], adaptations incurred during altitude training could be differentially affected by position-specific activities and their potential impact on individual oxygen saturation and internal hypoxic dose [18].

Garvican-Lewis et al. [19] reported on three normobaric ‘live high-train low’ (LHTL) interventions over a 6-month period, during which 11 field players of the Australian women’s water polo team engaged in passive hypoxic exposure (from 2500 to 3000 m simulated altitude), 14 h per day, over 9–11 days, while all training sessions were conducted at 580 m above sea level. Mean tHb_mass_ increments of 3.7 to 4.5% over the three altitude periods, as well as a large correlation (r = 0.73) between the players’ tHb_mass_ and performance in a multistage shuttle swim test, were reported [19]. However, to the best of the authors’ knowledge, no reports are available on the hematological consequences of natural altitude camps in world-class women’s water polo.

Considering the multitude of factors affecting altitude training adaptation [20,21], and the additive stress imposed by a demanding training camp and hypoxic environment, an effective daily monitoring system is needed to assess athletes’ fitness and fatigue responses to training, ensure sustained adaptation, and limit the risk of illness, injury, maladaptation, or overtraining during altitude training [4,11]. The aims of this case report investigation were three-fold: (i) to assess the hematological adaptations in a group of world-class female water polo players during a 16-day LHTH camp in the lead-up to a major international tournament; (ii) to evaluate whether hematological adaptations differed between players who were subsequently selected for the tournament and players not selected, as well as between playing positions; and (iii) to assess the interactions between markers of the athletes’ adaptation and the observed hematological changes.

## 2. Materials and Methods

### 2.1. Participants

The 22 world-class [22] female water polo players (3 goalkeepers, 15 field players, and 4 center forwards) participated in a 16-day altitude training camp. All players were preselected members of the Spanish national team preparing for the Gwangju 2019 FINA World Championships. Seventeen players had previous altitude training experience, although their last altitude camp had taken place two years before, and five players had never trained at altitude before.

### 2.2. Experimental Design

A single-group research design was implemented, as the world-class level of the participants precluded the implementation of a control group of similar caliber. The altitude camp was conducted at the Sierra Nevada High Performance Center (2320 m) in June 2019, finishing three weeks before the opening match of the Gwangju 2019 FINA World Championships. Training during the camp included 41 h of water polo-specific training, 5.5 h of swimming, 12 h of dryland strength training, 12 h of yoga and relaxation, and 6 h of mountain hiking. The players had 3 half-days and one full day of rest during the camp. All athletes ingested a daily dose of 42–100 mg of ferrous sulfate or ferrous glycine for the duration of the camp. All procedures were part of the team’s service provision, which conformed to the Code of Ethics of the World Medical Association (Declaration of Helsinki). Athletes provided informed consent to participate in monitoring procedures associated with team duties, with the understanding that data may be used for research purposes.

### 2.3. Procedures

#### 2.3.1. Total Hemoglobin Mass

The hematological measures tHb_mass_ and blood volume were measured at sea level 5 days before the start and on the last day of the camp, using an optimized carbon monoxide (CO)-rebreathing method [23]. The CO dose was 0.8 mL/kg body mass. The rebreathing procedure was performed for 2 min, in a seated position, through a glass spirometer (Blood tec GmbH, Bayreuth, Germany). Fingertip capillary blood samples (200 μL) were obtained immediately before the test, and 6 and 8 min following CO inhalation for determination of %HbCO (ABL80 FLEX CO-OX analyzer, Radiometer, Copenhagen, Denmark). Hemoglobin concentration (g/dL) and hematocrit (%) were determined in capillary blood (Hemo Control, EKF Diagnostics, Cardiff, UK). All measurements of tHb_mass_ and blood volume were performed by the same experienced researcher using the same equipment, with a standard error of 8.2 g and 27.9 mL in 25 duplicate measurements separated by 24 h.

#### 2.3.2. Blood Analyses

Two weeks before the camp and on the last morning of the camp, fasted forearm venous blood samples (15 mL) were collected to perform hematological analyses through standard procedures in an accredited clinical analysis laboratory. Briefly, the athlete reported to the same clinical analysis laboratory after overnight fasting. Blood was collected by venepuncture from an antecubital vein after 20 min of seated rest. The samples were analyzed for full hematology (flow cytometry, spectrometry, Sysmex XN, Sysmex Corporation, Kobe, Japan), including red cell count, differential leucocyte counts (neutrophils, lymphocytes, monocytes, eosinophils, and basophils), and iron status (molecular absorption spectrometry, immunoturbidimetry, cobas c 702, and cobas e 801 (Roche Diagnostics, Basel, Switzerland)), including serum iron, total iron-binding capacity, transferrin, saturation index, and ferritin.

#### 2.3.3. Daily Assessments

Daily assessment of players’ adaptations included resting heart rate (HR) and peripheral oxygen saturation upon waking (SpO_2_, Beurer PO 30, Ulm, Germany), body mass monitored after voiding (Seca 877, Hamburg, Germany), Rating of Fatigue [24], self-reported sleep hours, and subjective sleep quality using a Likert scale (5 = “very good”; 4 = “good”; 3 = “average”; 2 = “bad”; and 1 = “very bad”) [25]. Morning mid-stream urinary specific gravity (USG, UG-α Digital Refractometer, Atago Co., Tokyo, Japan) was assessed on four occasions during the camp.

### 2.4. Statistical Analysis

All analyses were conducted using R [26] (version 4.2.2, R Foundation for Statistical Computing, Vienna, Austria) in RStudio (version 2022.12.0 + 353, Posit Software PBC, Boston, MA, USA). Linear mixed models (LMMs) with a random intercept for each participant were used to determine the change in hematological variables from PRE to POST. Likewise, LMMs with a random intercept for each participant were used to determine differences between playing positions (goalkeepers, field players, and center forwards) and selection status (selected, n = 13 and non-selected n = 9) for hematological and daily assessment variables. The significance (α = 0.05) of main effects (*b*) was determined by a type II Wald F test. The Benjamini and Hochberg false discovery rate adjustment method was used in the case of multiple contrasts (i.e., playing positions).

Standardized effect sizes (ESs) were calculated using estimated marginal means and the pooled standard deviation of the random effects to account for the structure of the LMM. Standardized effect sizes were interpreted as trivial: |*d*| < 0.20, “small”: |*d*| 0.20–0.49, “moderate”: |*d*| 0.50–0.79, and “large”: |*d*| ≥ 0.80 [27]. A generalized additive mixed model (GAMM) with a random intercept for each participant was used to model the changes in daily assessment variables across the 16-day camp. “Periods of temporal change” in the daily assessment variables were detected via inspection of the GAMM first derivatives and their 95% simultaneous confidence intervals (with significance accepted when the simultaneous interval did not include 0) [28].

## 3. Results

The total hypoxic dose was 384 h or 891 km·h [8] excluding the 6 h of mountain hiking at variable altitudes ranging from 2320 to 3397 m. The pre-altitude ferritin values were 55 ± 28 ng/mL (range 12–117 ng/mL).

### 3.1. Hematological Adaptations

**Figure 1:**
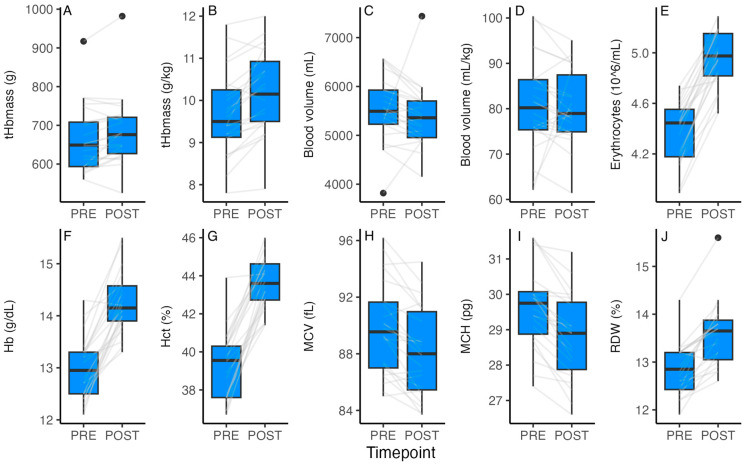
Hematological values before (PRE) and on last day (POST) of exposure to 2320 m of altitude. (**A**): absolute total hemoglobin mass (g) (*p* = 0.013, ES = −0.21); (**B**): relative total hemoglobin mass (g/kg) (*p* < 0.001, ES = 0.50); (**C**): absolute blood volume (mL) (*p* = 0.316,ES = 0.21); (**D**): relative blood volume (mL/kg) (*p* = 0.798, ES = 0.05); (**E**): erythrocytes (*p* < 0.001, ES = 2.48); (**F**): hemoglobin concentration (*p* < 001, ES = 2.14); (**G**): hematocrit (*p* < 0.001, ES = 2.69); (**H**): mean corpuscular volume (*p* < 001, ES = 0.51); (**I**): mean corpuscular hemoglobin (*p* < 0.001, ES = 0.72); and (**J**): red cell distribution width (*p* < 0.001, ES = 1.21). Grey lines show PRE and POST values for individual participants.

The tHb_mass_ increased from PRE to POST when expressed as relative (g/kg) (5.4 ± 5.1%, range −3.9 to 20.2%; *p* < 0.001, ES = −0.50, Figure 1) and absolute (g) values (2.9 ± 5.1%, range −6.3 to 15.5%, *p* = 0.013, ES = −0.21). Blood volume did not change when expressed as relative (mL/kg) (*p* = 0.797, ES = 0.05) or absolute (mL) values (*p* = 0.316, ES = 0.21). The change in tHb_mass_ during the altitude camp was not associated with the pre-altitude tHb_mass_ (*b* = −0.13, 95%CI = −0.34 to 0.08, R^2^ = 0.07, *p* = 0.218) or ferritin concentration (*b* = −0.005, 95%CI = −0.012 to 0.003, R^2^ = 0.08, *p* = 0.205). The erythrocyte count, hemoglobin concentration, hematocrit, and red cell distribution width also increased from PRE to POST (*p* < 0.001, ES = 1.21–2.69), while mean corpuscular volume and mean corpuscular hemoglobin decreased (*p* < 0.001, ES = 0.51 and 0.72, respectively; Figure 1). No substantial differences were identified in any of the hematological parameters between selected and non-selected players. There was a large difference in blood volume relative to body mass between the centers (PRE 74.1 ± 5.4 and POST 69.7 ± 5.9 mL/kg; mean ± SD) and field players (PRE 80.8 ± 10.6 and POST 82.8 ± 6.8 mL/kg; adj *p* = 0.046, ES = 1.15), and between centers and goalkeepers (PRE 89.7 ± 9.6 and POST 82.0 ± 7.1 mL/kg; adj *p* = 0.046, ES = 1.62).

### 3.2. Daily Assessments of Players’ Adaptations

**Figure 2:**
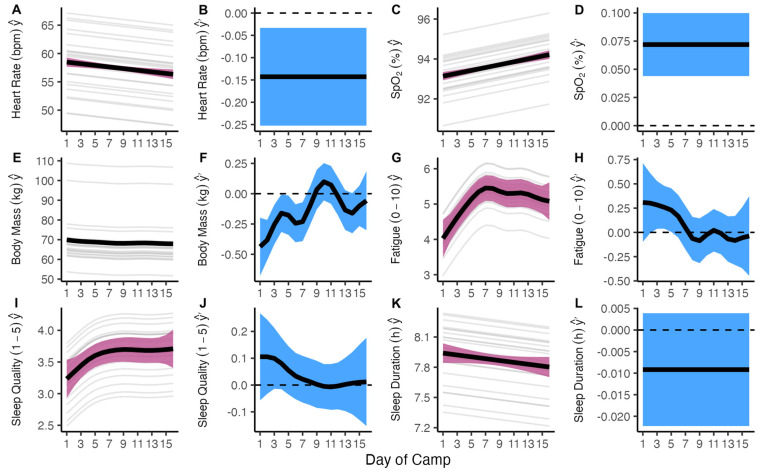
Changes in daily assessment variables across 16-day camp. Model estimates ($\hat{y})$ and simultaneous 95% confidence intervals are expressed at population level (black line and pink ribbon), and participant level (grey lines). First derivative estimates $\left({\hat{y}}^{\prime }\right)$ and their simultaneous 95% confidence intervals (black line and blue ribbon) show the rate of change in the response variable at a given timepoint. (**A**,**B**): resting heart rate; (**C**,**D**): resting peripheral oxygen saturation; (**E**,**F**): body mass; (**G**,**H**): Rating of Fatigue; (**I**,**J**): sleep duration; and (**K**,**L**): sleep quality.

The time course of the daily markers of players’ adaptations for the duration of the camp is presented in Figure 2. Resting heart rate declined from 59 bpm (SD = 6.3) to 57 bpm (SD = 9.2) from day 1 to day 16 of the camp (d = 0.28 by 0.14 bpm each day [95% CI = −0.55 to −0.22]). SpO_2_ increased from 93.0 (SD = 1.4) to 94.2 (SD = 1.8) (d = 0.74 by 0.07 percentage points each day [0.04–0.10]). Body mass declined by 0.16 (−0.32 to −0.01) to 0.44 (−0.68 to −0.20) kg per day for the first seven days of the camp. A small difference (*p* = 0.039, ES = 0.49) in sleep duration was observed between the selected (8.0 h, SD*_w_* = 0.5, SD*_b_* = 0.3) and non-selected players (7.7 h, SD*_w_* = 0.6, SD*_b_* = 0.4). Large differences were observed in the body mass between centers (89.3 kg, SD*_w_* = 1.0, SD*_b_* = 16.3) and field players (64.9 kg, SD*_w_* = 0.7, SD*_b_* = 2.9) (adj *p* < 0.001, ES = 3.37) and between centers and goalkeepers (59.0 kg, SD*_w_* = 0.5, SD*_b_* = 6.0) (adj *p* < 0.001, ES = 4.19). Large standardized differences were also evident for USG between centers (1.023, SD*_w_* = 0.003, SD*_b_* = 0.003) and field players (1.019, SD*w* = 0.003, SD*_b_* = 0.003) (adj *p* = 0.036, ES = 1.15), where SD*_w_* = standard deviation within players, and SD*_b_* = standard deviation between players.

## 4. Discussion

A well-structured and supported 16-day altitude training camp undertaken by a national women’s water polo team elicited substantial increases in absolute and relative tHb_mass_, erythrocytes, hemoglobin concentration, and hematocrit. There were only trivial differences in the hematological parameters between selected and non-selected players for the World Championships held 3 weeks after the altitude camp. Notwithstanding the limitations of a single-group research design, these data appear to confirm the utility of an altitude training camp to facilitate hematological adaptations in world-class level team-sport (in this case water polo) players prior to international competition. Although the objective measures of players’ post-altitude fitness or performance level were not collected in this study, daily markers of adaptation during the camp allowed for the modulation of training loads to facilitate a positive adaptation. In this respect, it is worth mentioning that the thirteen selected players went on to win the silver medal at the Gwangju 2019 FINA World Championships, whereas seven of the non-selected players also won the silver medal at the inaugural beach water polo tournament that took place at the same World Championships.

### 4.1. Hematological Adaptations

The 16-day LHTH camp elicited positive hematological changes, including increased absolute and relative tHb_mass_, erythrocyte count, hemoglobin concentration, and hematocrit. The increments in tHb_mass_ would likely result in improved aerobic power [29] and water polo-specific fitness [19]. The observed 2.9% increase in absolute tHb_mass_ is close to that predicted by the linear model of Garvican-Lewis et al. [8] (2.97% based on a hypoxic dose of 891 km·h), but was somewhat lower than the predicted values from the quadratic and exponential models (3.51% and 3.54%, respectively) and the values reported for international caliber endurance athletes undergoing a higher altitude dose (3.4% tHb_mass_ increase for a hypoxic dose of 989 km·h) [30]. Relative to body mass, the ~5% increase in tHb_mass_ was larger than previously reported following LHTL in a similar athlete cohort, probably due to the larger hypoxic dose in the present study (384 h vs. 150–160 h) [19]. An increase in tHb_mass_ would likely promote both the aerobic (directly via compensatory improvements in oxidative metabolism) and anaerobic capacities (indirectly via improved capacity to tolerate high intensity interval training) required in water polo match play [17]. The clinical significance of the changes in mean corpuscular volume, mean corpuscular hemoglobin, and red cell distribution width, if any, remains to be elucidated.

It has been suggested that no single altitude training recommendation can be implemented across team sports or for all players in a team, and that individualized hypoxic dose and modality recommendations would be required for optimal altitude training outcomes [11]. In the present study, all players were exposed to the same “external” hypoxic dose, and although no substantial differences were observed between the selected and non-selected players, nor between playing positions, these outcomes may have been influenced by the small sample sizes. Thus, some variable individual hematological responses were apparent (see Figure 1), and two of the three goal keepers showed a decrease in tHb_mass_ during the altitude camp. Whether the individual differences were related to the different “internal” hypoxic dose (saturation hours) remains to be elucidated [18]. Moreover, the lower relative blood volume of centers compared to field players and goalkeepers was likely related to their substantially larger body mass. The lack of a difference between the selected and non-selected players suggests that coaches prioritize fitness factors, such as speed and power, and/or technical and tactical abilities to make decisions on team composition; the similarity of adaptations between playing positions implies that all players could benefit from altitude training, despite positional differences in match activity profiles [13,16].

Recent research suggests that high-intensity interval training in hypoxia may be more effective at improving maximal oxygen uptake than similar training performed in normoxia [31]. In addition, elite female rugby sevens players’ repeated sprint performance has been shown to improve after superimposing repeated sprints in hypoxia during 3 weeks of LHTH [32]. Recent reports also indicate the potentially positive interactions between resistance training in hypoxic conditions and strength and power outcomes [33,34,35]. In this sense, it is worth mentioning that although a repeated sprint training protocol as such was not implemented, the ~46 h of water polo-specific and swimming training in the present group of players involved large amounts of high-intensity interval training, as well as 12 h of dryland resistance training. Based on the above evidence, it is possible that the hypoxic environment could have contributed to enhanced training adaptations.

### 4.2. Daily Markers of Adaptation

A detailed longitudinal monitoring of athletes’ physical and perceptual responses during altitude training is recommended to maximize the benefits of altitude training and minimize the risk of maladaptation [4]. In this study, the recommended markers and tools were selected to monitor athletes’ adaptation and manage day-to-day training [4,25]. In keeping with previous reports on elite swimmers and professional cyclists [25], the resting heart rate and body mass declined, whereas SpO_2_ increased throughout the camp (Figure 2), evidence of a positive adaptation to both the hypoxic environment and the training program. These results, however, are in contrast with a recent investigation on the French national female rugby sevens team completing a 3-week LHTH camp 1850 m, in which no substantial changes were observed in the body mass, morning HR, and SpO_2_ from PRE to POST altitude training [32].

Sleep is an essential component of athletes’ recovery and adaptation processes [36]. However, sleep is often impaired during altitude training, which may have a negative impact on athlete’s recovery, adaptation, and subsequent performance [37,38]. A recent investigation on the sleep characteristics of elite endurance athletes during a 3-week LHTH camp at 1800 m reported a reduced total sleep time and light sleep compared to sea level, as well as increased deep sleep, which was associated with increased SpO_2_ [37]. In the present study, self-reported sleep quality increased during the first week of the camp (Figure 2), while sleep duration remained quite constant throughout the camp. Moderate and most likely spurious associations were detected between sleep duration and the changes in tHb_mass_ and blood volume. In addition, a small difference in sleep duration (~20 min) was observed with selected players having longer sleep than non-selected players. It is unclear whether a difference of this magnitude has a substantial effect on the physical and/or mental factors underpinning training and competition performance, but the cumulative effects of day-to-day variations in sleep duration of just ~8 to 12 min could have a substantial influence on subjective recovery, adaptations, and performance optimization over the duration of an altitude camp [37].

### 4.3. Limitations

Several limitations should be declared. We are aware that a single-group research design poses a threat to the internal validity and the generalizability of the findings and makes it challenging to establish causality. In this instance, the design precludes the clear differentiation of the effects of altitude compared with those related to the training program. However, utilizing a formal control group at sea level is usually not an option when working with a (small and/or single) squad of world-class athletes. In addition, a much larger cohort would be required to adjust for the potential covariates (e.g., age and body composition). Despite its limitations, this type of research design is characterized by its simplicity and cost effectiveness and is useful for initial exploration and providing baseline information for future research.

Neither the direct impact of altitude training on non-hematological altitude adaptations nor on individual players’ fitness and actual competition performance 3 weeks after the altitude camp could be assessed from the present investigation. To this end, pre- and post-altitude camp sport-specific fitness and performance testing would have been required. Recent studies have shown conflicting results regarding the maintenance of hematological adaptations and their potential impact on performance upon return to sea level: Astridge et al. [39] observed an increased tHb_mass_ but no evidence of improved sea level performance in a group of high-performance swimmers 2 weeks post-altitude, whereas Krumm et al. [40] reported a swift loss of tHb_mass_ within 2 weeks after altitude, but excellent performance outcomes at world championships 3 weeks post-altitude in elite to world-class racewalkers. Furthermore, both acute and chronic hematological responses to hypoxia are known to be sensitive to fluctuations in sex hormones [41]. Although tHb_mass_ does not seem to be affected by menstrual cycle phase, relative tHb_mass_ can increase in women using oral contraceptives in comparison to non-users [42]. In the present investigation, participants’ self-reported information regarding their menstrual cycle was collected at the time of testing, but the potential impact of the menstrual phase on altitude-induced adaptations could not be evaluated.

### 4.4. Practical Applications

World-class and elite water polo players could benefit from strategically planned ‘live high-train high’ altitude training camps in the lead-up to major international tournaments. The hematological adaptations demonstrated here could positively impact various fitness traits associated with water polo match performance, and this appears to be irrespective of playing position. In this respect, performing standardized pre- and post-altitude fitness and performance testing is recommended to assess the sport-specific implications of the observed hematological changes. The daily monitoring of players’ adaptation is recommended to guide training and avoid maladaptation to the combined stress imposed by a demanding training camp and the hypoxic environment.

## 5. Conclusions

Within the limitations of a single-group research design, a well-structured and sports science-supported altitude training camp over 16 days can induce favorable hematological adaptations in world-class women’s water polo players. There were no substantial differences in the hematological parameters between selected and non-selected players. However, field players showed larger increases in blood measures than centers and goalkeepers over the 16 days. The present results also underscore the relevance of the adequate daily monitoring of athletes’ adaptation (e.g., heart rate, SpO_2_, fatigue, iron status, hydration, and sleep) to maximize the potential benefits of altitude training camps. Although the present results should be interpreted with caution, this form of training camp can be useful in preparing team-sport players for international competitions.

## Data Availability

The datasets generated during this investigation are available from the corresponding author on reasonable request.
